# Clinical analysis of subxiphoid vs. lateral approaches for treating early anterior mediastinal thymoma

**DOI:** 10.3389/fsurg.2022.984043

**Published:** 2022-09-09

**Authors:** Bin Li, Lijuan Niu, Chenqi Gu, Kaiwen He, Ruizhi Wu, Zhenfeng Pan, Shaomu Chen

**Affiliations:** ^1^ Department of Thoracic Surgery, The First Affiliated Hospital of Soochow University, Suzhou, China; ^2^ Department of Endocrinology, The First Affiliated Hospital of Soochow University, Suzhou, China; ^3^ Department of Imaging, The First Affiliated Hospital of Soochow University, Suzhou, China; ^4^ Department of Thoracic Surgery, Changshu No.1 People's Hospital, Changshu, China; ^5^ Department of Pharmacy, The First Affiliated Hospital of Soochow University, Suzhou, China

**Keywords:** Masaoka stage system, visual analogue scale pain score, anterior mediastinal thymoma, transthoracic video-assisted thoracoscopic thymectomy, subxiphoid video-assisted thoracoscopic thymectomy

## Abstract

**Objective:**

To investigate the clinical efficacy of the subxiphoid approach for early anterior mediastinal thymoma and evaluate its advantages over the lateral intercostal approach.

**Methods:**

A total of 345 patients with early anterior mediastinal thymoma were retrospectively analyzed from January 2016 to December 2020 in the First Affiliated Hospital of Soochow University. Out of these, 99 patients underwent subxiphoid video-assisted thoracoscopic thymectomy and 246 patients underwent transthoracic video-assisted thoracoscopic thymectomy. We compared the intraoperative conditions (such as operation time and intraoperative blood loss), postoperative conditions [such as postoperative pleural drainage volume, extubation time, postoperative hospital stay, and postoperative visual analogue scale (VAS) pain score], and postoperative complications (such as death, pneumonia, delayed wound healing, cardiac arrhythmia, and phrenic nerve injury) of the two groups and analyzed the clinical advantages of the subxiphoid approach for treating early anterior mediastinal thymoma.

**Results:**

There was no significant difference between the two groups in terms of general clinical features, operation time, and postoperative complications (*P* > 0.05).However, there was a significant difference in terms of intraoperative blood loss, postoperative pleural drainage volume, tube extubation time, postoperative hospital stay, postoperative VAS pain score, and postoperative analgesics (a significantly decreased flurbiprofen axetil amount) (*P* < 0.05).

**Conclusion:**

Compared with the lateral intercostal thoracic approach, the subxiphoid approach had advantages in terms of intraoperative blood loss, postoperative hospital stay, tube extubation time, postoperative pleural drainage volume, postoperative VAS pain score, and analgesics dosage. It could provide a better view of the bilateral pleural cavities and more thorough thymectomy and superior cosmesis, and it proved to be a safe and feasible minimally invasive surgical method.

## Introduction

Thymoma is the most common lesion in the anterior mediastinum. At present, the Masaoka stage system proposed in 1981 is used in pathological classification, and the Masaoka–Koga stage system proposed by the Japanese scholar Masaoka is used in clinical classification (Table 1) (1). Thoracectomy has always been considered the best treatment for anterior mediastinal thymoma, and complete resection is the most important factor that significantly affects postoperative long-term overall survival (2). Thoracectomy should include complete exposure of the entire anterior mediastinal vision to facilitate complete removal of the entire thymus and mediastinal fatty tissue. A study conducted by Nguyen et al. (3) showed that the 10-year survival rate of type A thymoma after complete thymus resection was 97%, 95% for AB, 92% for B1, 81% for B2, 62% for B3, and 29% for thymic carcinoma. The midline sternotomy approach was recognized as the gold standard for a complete thymectomy. Video-assisted thoracoscopic surgery (VATS) was first reported in 1993, and it had advantages such as less operative blood loss, less damage to surrounding tissue, and reduced postoperative pain and complications compared with the traditional midline sternotomy approach. Resection of the large thymus and complicated procedures are contraindicated to VATS, and minimally invasive surgery for resection of the large thymus could not guarantee a complete resection of ectopic thymic tissue (4). In recent years, VATS has emerged as a highly popular surgery. The VATS method includes subxiphoid video-assisted thoracoscopic thymectomy (S-VATT) and transthoracic video-assisted thoracoscopic thymectomy (T-VATT). T-VATT is divided into right thoracoscopic (RtT) and left thoracoscopic (LtT) approaches. These two approaches have no significant difference in terms of clinical efficacy, but the RtT approach is more common due to its providing a better surgical vision. After conducting a meta-analysis, Li et al. (5) found that the operative time, intraoperative blood loss, hospital stay time, and postoperative complications of patients in the S-VATT group were significantly reduced compared with those in the T-VATT group, while there was no significant difference in terms of the postoperative thoracic drainage tube placement time, conversion to open approach, and oncologic outcomes. L-VATT group need to face worse surgical vision of mediastinal fatty tissue and contralateral phrenic nerve, and it is almost difficult to completely remove the contralateral mediastinal fatty tissue, which often requires more incision and increases the trauma of the operation. In general, the subxiphoid approach produces shorter operation time, less intraoperative blood loss, shorter hospital time, superior cosmetic outcomes, and fewer postoperative complications. In this study, we enrolled 345 patients who underwent resection of the entire thymus by VATS from January 2016 to December 2020 in the Department of Thoracic Surgery of First Affiliated Hospital of Soochow University in January 2020 and then compared the operational situations between the two groups, so as to test the clinical efficacy of the subxiphoid approach compared with that of the lateral intercostal thoracic approach.

**Table 1:** Masaoka–Koga staging system.

Installments	Performance
Phase I	The envelope was complete and no tumor cells invaded the envelope
Phase II a	Microscopically, the tumor cells invaded the envelope
Phase II b	Tumor cells infiltrated into the pleural or adipose tissue
Phase III	Tumor cells invaded adjacent organs such as the lungs, pericardium, large blood vessels, and more
Phase IVa	The tumor occurred with implant metastases to the pleural or pericardium
Phase IVb	Tumor cell metastases through the lymphatic tract or blood, with distant metastasis

## Methods

### Inclusion/ exclusion criteria

Inclusion criteria: (1) Confirmation of preoperative anterior mediastinal mass, lesion diameter of ≤5 cm, and no vascular and peripheral tissue invasion; (2) Phase I–II according to the Masaoka stage and no metastatic lesions;

Exclusion criteria: (1) Patients who were converted to the open approach during the surgery; (2) Patients with severe myasthenia gravis; (3) Patients who received palliative surgery for incomplete resection because of intraoperative bleeding or the long diameter of the thymus >5 cm; (4) Preoperative imaging indications of vascular or peripheral tissue invasion or distant metastasis according to postoperative pathology; (5). Those who used opiates preoperatively or with a history of chronic pain syndrome.

### Surgical method

#### Subxiphoid approach

Anesthesia method: The patient was given general anesthesia using single-cavity tracheal intubation to provide mechanical ventilation.

Surgical position: supine, legs open in scissors, surgeon standing between the patient’s legs with the assistant on the right side.

Incision selection: A 2-cm observation hole was created below the lower edge of the xiphoid, the rectus abdominis muscle was separated, and space was created bluntly with the surgeon’s finger to extend the working gap. Two 0.5-cm incisions were created under the bilateral rib bow as the operating holes.

A 1-cm, 30° oblique rigid thoracoscope was inserted through the observation hole. An 8–10 cm H_2_O-positive pressure of carbon dioxide (CO_2_) was injected into the anterior mediastinum to establish artificial pneumothorax and expand posterior sternum clearance. The ultrasonic scalpel and thoracoscopic clamps were inserted into the bilateral operating holes.

#### Lateral intercostal thoracic approach

Anesthesia method: General anesthesia was used, double-lumen endotracheal intubation was used instead of single-lumen tracheal intubation, and unilateral single pulmonary ventilation was employed.

Surgical position: The patient was placed at 30–45° in a semisupine position.

#### Surgical steps

The free anterior edge of the thymus gland along the posterior sternum space was gradually dissected vertically toward the thoracic outlet and laterally free toward the bilateral diaphragm attachment point. Simultaneously, adipose tissue of the bilateral diaphragmatic angle was dissected. The mediastinal pleural membrane was incised on both sides, avoiding paradoxical injury during the operation. It was necessary to carefully identify the bilateral diaphragm nerves to avoid causing postoperative hiccups, diaphragm disorders, and other surgical complications.

At the same time, it was necessary to carefully distinguish the surrounding environment during the dismemberment of the thymus vein, which can be gradually sharpened and blunt along the beginning of the nameless vein to the distal end, fully free from the thymus vein and cut off after the titanium clamp is closed. The anterior pericardial fat pads and fat pads at the diaphragm angle and the main lung window were both freed using a harmonic ultrasound knife, and the entire thymus and adipose tissue that were completely removed were collected in the retrieval bag through the subxiphoid observation hole.

After the operation was completed, the substabular observation hole was routinely inserted into a mediastinal drainage tube. During the operation, attention was paid to the complete removal of the thymus tissue and anterior mediastinal fat, the upper boundary to the nameless vein, the lower boundary to the supraphragm pericardium, and the bilateral to the diaphragm nerve. In order to ensure the integrity of the capsule and the complete detachment of the tumor, the surgeon carefully grabbed the normal tissue but did not contact the tumor capsule. Specimens were sent for examination.

#### Right thoracoscopic approach

Generally, a 1.5 cm incision is selected between the sixth intercostal space of the right midaxillary line as the observation hole and a trocar is inserted. A 3-cm incision is made as the main operation hole in the right fourth intercostal space of the posterior axillary line, and the incision position can be adjusted according to the operational situation. A 1.5-cm incision is made at the sternum with the trocar as the secondary operation hole.

Here, an ultrasound knife was used to open the mediastinal pleura along the back of the internal thoracic arteriovenous vein, the superior vena cava, and the front of the right diaphragm nerve; dissected carefully; the free anterior mediastinal fat and the right lobe of the thymus were exposed; the lower right pole of the thymus at the base of the heart was bluntly and sharply isolated; the right lobe of the thymus to the right posterior was pulled; and the left lobe of the thymus was separated from the lower left pole. The thymus gland was lifted upward, the thymus vein was isolated at the angle of the vein, the titanium clamp was closed and cut off, and the upper right pole of the thymus gland was completely separated. The thymus tissue was pulled out, the upper left pole was separated, and the thymus tissue was completely removed.

#### Left thoracoscopic approach

Usually, a 1.5-cm incision is made between the fifth rib of the left axillary midline as the observation hole and the trocar is inserted, a 3 cm incision is selected between the third intercostal of the left midaxillary line, and a 1.5 cm incision is made between the fifth intercostal of the clavicle midline as the main and secondary operation holes.

Here, the mediastinal pleura was opened behind the arteriovenous veins in the thoracic cage and in front of the phrenic nerve. The ultrasound knife separated the anterior mediastinal fat and thymus tissue, sharply combined with a blunt separation of the lower left pole of the thymus from the anterior edge of the heart, pulled the left lobe of the thymus together with the mediastinal pleura backward and downward, and separated the right lobe of the thymus from the lower right pole. The ultrasound knife separated the left upper pole of the thymus along the nameless vein, it separated the thymic vein, and cut it off after titanium clamps to close the thymic vein. The upper right pole of the thymus gland was pulled to the lower left and the Ultrasonic knife to separate thymus.

### Observation indicators

(1) General information: the patient's age, gender, and body mass index (BMI);
(2) Tumor condition: the maximum diameter of the lesion, postoperative pathological type, and clinical stage;
(3) Perioperative-related indicators: operation time, intraoperative blood loss, total postoperative pleural drainage volume, postoperative extubation time, the total number of hospital stay days after surgery, postoperative visual analogue scale pain score on the first day after surgery, the total dosage of postoperative analgesics, and perioperative complications.

### Statistical processing

The continuous variables are represented as the mean ± standard difference, and the independent sample *t*-test is used to compare the normal distribution variables; the Mann–Whitney *U* test compares the non-normal distribution variables. The classification variables are represented as a percentage, and the differences between groups are compared using card-square tests. All statistical analyses were performed using SPSS software (SPSS version 20.0), and *P* < 0.05 indicated a statistical difference.

## Results

A total of 345 patients with early-stage anterior mediastinal thymoma were selected from the Department of Thoracic Surgery of the First Affiliated Hospital of Soochow University from January 2016 to December 2020. The postoperative clinical stages of all patients were between stage I and II according to the Masaoka stage. Of these, 99 patients underwent S-VATT, and 246 underwent T-VATT. Of the 99 patients in the S-VATT group, 51 were males, 48 were females, aged 49.05 ± 12.56 years, and the tumor size was 3.74 ± 0.76 cm. Of the 246 patients in the T-VATT group, 110 were males, 136 females, aged 50.69 ± 13.89 years, and the tumor size was 3.72 ± 1.09 cm. A complete thymus resection was performed in all patients.

The general clinical features of the two groups are given in Table 2. There are no statistical differences in terms of age, sex, BMI, the diameter of the lesion, Masaoka stage, and WHO stage (*P *> 0.05).

**Table 2:** Comparison of the clinical characteristics between the two groups.

	S-VATT (*n* = 99)	T-VATT (*n* = 246)	*P*-value
Age (years of age)	49.05 ± 12.56	50.69 ± 13.89	0.310
Gender			0.252
Male	51	110	
Female	48	136	
BMI (kg/m^2^)	24.35 ± 3.48	24.26 ± 3.90	0.834
Diameter (cm)	3.74 ± 0.76	3.72 ± 1.09	0.876
Masaoka stage			0.885
Phase I	31	79	
Phase II	68	167	
WHO stage			0.785
Type A	13	31	
Type AB	28	69	
Type B1	23	72	
Type B2	18	41	
Type B3	17	33	

S-VATT, subxiphoid video-assisted thoracoscopic thymectomy; T-VATT, transthoracic video-assisted thoracoscopic thymectomy; BMI, body mass index.

The intraoperative situations of the S-VATT and T-VATT groups are given in Table 3. All operations were performed under thoracoscopy without thoracotomy. There was no statistical difference in mean surgical time (97.83 ± 29.05 vs. 98.71 ± 42.47, *P* = 0.825). However, intraoperative blood loss (32.02 ± 41.48 vs. 58.81 ± 82.01, *P *< 0.01) in the S-VATT group was significantly higher than that in the T-VATT group.

**Table 3:** Comparison of intraoperative indicators between the two groups.

	S-VATT (*n* = 99)	T-VATT (*n* = 246)	*P*-value
Operation Time (min)	97.83 ± 29.05	98.71 ± 42.47	0.825
Interoperative blood loss volume (ml)	32.02 ± 41.48	56.87 ± 82.01	<0.01

S-VATT, subxiphoid video-assisted thoracoscopic thymectomy; T-VATT, transthoracic video-assisted thoracoscopic thymectomy.

The postoperative indicators of the two groups are given in Table 4. In the S-VATT group, the postoperative pleural drainage volume (240.03 ± 169.54 vs. 359.01 ± 461.74, *P* = 0.001.001), extubation time (2.77 ± 0.82 vs. 3.53 ± 1.94, *P *< 0.01), and postoperative hospital stay days (3.75 ± 1.02 vs. 4.40 ± 2.09, *P *< 0.01) were better than those in the T-VATT group.

**Table 4:** Comparison of postoperative indicators between the two groups.

	S-VATT (*n* = 99)	T-VATT (*n* = 246)	*P*-value
Postoperative pleural drainage volume (ml)	240.03 ± 169.54	359.01 ± 461.74	0.001
Extubation time (d)	2.77 ± 0.82	3.53 ± 1.94	<0.01
Postoperative hospital stay (d)	3.75 ± 1.02	4.40 ± 2.09	<0.01
Day 1 after surgery (VAS)	3.12 ± 0.70	4.59 ± 0.79	<0.01
Flurbiprofen axetil (mg)	228.28 ± 148.49	269.11 ± 154.20	0.025

S-VATT, subxiphoid video-assisted thoracoscopic thymectomy; T-VATT, transthoracic video-assisted thoracoscopic thymectomy; VAS, visual analogue scale.

Patients in the S-VATT group had less pain than those in the T-VATT group. The first-day VAS score (3.12 ± 0.70 vs. 4.59 ± 0.79, *P* < . 0.0.01) and the use of flurbiprofen axetil (228.28 ± 148.49 vs. 269.11 ± 154.20, *P* = 0.025) were better in the S-VATT group than in the T-VATT group.

A comparison of postoperative complications in the two groups is given in Table 5. There were no deaths of patients in the two groups. The total incidence of perioperative complications in both groups was 11.1% vs. 7.3%, *P* = 0.71, respectively, with no statistical differences. In the S-VATT group, one patient was diagnosed with postoperative pneumonia, six patients had delayed wound healing, two had arrhythmias (paroxysmal atrial fibrillation and tachycardia), and two had phrenic nerve injury. In the T-VATT group, five patients had pneumonia, ten had delayed wound healing, two had arrhythmias, and one had phrenic nerve injury. Because of the small number of occurring cases, no statistical comparison was made.

**Table 5:** Comparison of postoperative complications in the two groups.

	S-VATT (*n* = 99)	T-VATT (*n* = 246)	*P*-value
Death	0	0	
Postoperative complications	11 (11.1%)	18 (7.3%)	0.71
Pneumonia	1	8	
Late wound healing	3	3	
Arrhythmia	2	5	
Phrenic nerve damage	5	2	

S-VATT, subxiphoid video-assisted thoracoscopic thymectomy; T-VATT, transthoracic video-assisted thoracoscopic thymectomy.

## Discussion

This study compares the advantages and disadvantages of the two methods, intraoperatively and postoperatively. As for the intraoperative situation, the study compared the operative time and the intraoperative blood loss volume, which showed that there was no difference between the two approaches, while the subxiphoid approach had a significant advantage in terms of reduction of intraoperative blood loss (32.02 ± 41.48 vs. 56.87 ± 82.01). This was consistent with the conclusions reached by Zhao et al. (6). The subxiphoid approach revealed advantages in terms of all the postoperative conditions conducted in this experiment, showing less postoperative pleural drainage volume, shorter extubation time and postoperative hospital stay days, and less postoperative pain. Chen et al. (7) found that the subxiphoid approach resulted in less postoperative pain or paralysis and more esthetic postoperative incisions without causing damage to the intercostal nerve. There was no difference between the two approaches in terms of postoperative complications because both approaches were minimally invasive, with fewer chest trauma and postoperative complications.

Before the emergence of thoracoscopy, the standard procedure of thoracectomy had always been the median sternotomy approach, which was first proposed by Blalock et al. (8) and his team in 1939 for the treatment of thymoma combined with severe myasthenia gravis. Although this procedure has an increased probability of postoperative mediastinal infection, postoperative pain, and longer postoperative recovery time, median sternotomy has always been considered the most effective method of thymectomy due to the complete removal of thymus and mediastinal adipose tissue (9). Since 1990, with the development of thoracoscopic techniques, the VATS method has been applied to the enlarged thoracectomy (10, 11). There are two approaches of the lateral intercostal thoracic approach: the RtT approach and the LtT approach. Pennathur et al. (12) found that the right thoracoscopic approach produced better surgical vision and could expose the innominate vein and thymus vein, so it became a hit with surgeons. The left thoracoscopic approach affected surgical vision and operating space due to the presence of the heart. The subxiphoid approach was first used by Suda et al. (13) and his team to treat a patient with myasthenia gravis. In addition, Suda introduced CO_2_ injection to facilitate the operation. Hsu et al. (14) conducted a retrospective analysis of 27 patients with myasthenia gravis, with 12 subjected to the right thoracoscopic approach and 15 subjected to the subxiphoid approach. The results were that the subxiphoid approach produced better bilateral phrenic nerve vision, more complete thymus resection, and the weight of the lesion was larger than that of the lateral thoracic approach. The subxiphoid approach produced a shorter learning curve and safer surgical methods. Zhang et al. (15) compared the perioperative outcomes of the subxiphoid approach and lateral intercostal thoracic approach for the treatment of thymoma, including clinical and surgical outcomes, postoperative pain scores, and cosmetic outcomes. The advantages of the subxiphoid approach include lower postoperative visual analogue scale pain score, shorter hospital stay days, decreased inflammatory cytokine response, and superior cosmesis. Postoperative complications between the two groups were not statistically significant and there were no perioperative deaths in both groups. The other advantages of the subxiphoid approach include adequate surgical vision of the cervical and bilateral thoracic cavity, reduced postoperative pain, and better cosmetic results. Decreased amounts of leukocytes and C-reactive protein were found in the subxiphoid approach group of patients. Xu et al. (4) found that there were less intraoperative blood loss, shorter chest tube extubation time, lower postoperative pain scores, shorter postoperative hospitalization time, and shorter surgical time in the subxiphoid group than in the lateral intercostal thoracic group. There were many disadvantages in the lateral intercostal thoracic approach, such as a worse vision of mediastinal fat exposure, more damage to the lateral phrenic nerve and intercostal nerve, and incomplete removal of the upper pole of the thymus. Compared with the other subxiphoid approach with holes, two additional subcostal operating holes avoid mutual interference of the device and make the procedure relatively simple. As for common thymectomy, there is no significant difference in surgical time between the two methods. When the thymus and anterior mediastinal fat are generally removed, the operation time of the subxiphoid approach is significantly lower than that of the thoracic approach. In a retrospective clinical study conducted by Yano et al. (16), the lateral intercostal thoracic approach was applied on 46 patients and the right thoracoscopic approach on 14 patients. The subxiphoid approach resulted in shorter surgery time, less chest trauma, and less bleeding than the lateral intercostal thoracic approach. Postoperative complications, the scope of the mass resection, the extubation time of the chest drainage tube, postoperative hospital stay time, and overall survival were not significantly different in the two groups. In addition, the lateral intercostal thoracic approach usually needed three holes, while the subxiphoid approach required only two holes. Phrenic nerve paralysis is most commonly seen in postoperative complications of the lateral intercostal thoracic approach. There were lesser skin trauma, lower white blood cells, and C-reactive protein counts in the lower group. The vision of those subjected to the subxiphoid approach is similar to those subjected to the midline sternotomy approach, which is commonly used by surgeons. The subxiphoid approach also solves the device interference problem, which exists in the lateral intercostal thoracic approach. Suda et al. (17) divided 81 patients who underwent anterior mediastinal mass removal into two groups: the VATS group, with a total of 35 people, and the subxiphoid thoracoscopy (SPT) group (subxiphoid approach), with a total of 46 people. There was no difference in surgical time between the two groups (*P* = 0.0853). Those in the VATS group had higher bleeding volume, longer hospitalization periods, and more postoperative analgesia medication than those in the SPT group. S-VATT was considered a safe surgical procedure with less trauma. This was consistent with the conclusion reached by Wu et al. (18).

## Outlook

Recent years have seen the emergence of robot-assisted subxiphoid approach thoracectomy. In 2004, Bakker et al. (19) performed robot-assisted subxiphoid approach thoracoscopy in 12 pigs. By pulling the sternum up and creating the three operating holes between the lower and the left fourth ribs, the entire thymus and the anterior mediastinal fatty tissue were completely removed. This demonstrates that robot-assisted subxiphoid approach thymectomy is safe and feasible. Ishikawa et al. (20) successfully conducted an enlarged thymectomy on a fresh human body in 2010, assisted by the Da Vinci robot system. Good bilateral phrenic vision and operating space for the nerve were obtained by hanging the sternum up. Robotic assistance provides a free space of the surgical device and a three-dimensional display of the surgical field. Robotic instruments have a considerable advantage over standard endoscopic instruments when separating the tissues of the phrenic nerves and major mediastinal veins such as the venae cava superior and innominate vein. Three robot-assisted subxiphoid approach thoracectomy procedures, conducted by Suda et al. (21), showed that by using the robot at a correct exposure angle, no postoperative complications resulted, demonstrating that robot-assisted thoracectomy is a promising new technique. Usually, the establishment of a surgical incision depends on the blunt separation of the fingers, which may lead to slow bleeding and then affect surgical vision. Xu and Zhang (22) introduced a new method for establishing surgical space into the retrosternal space by placing a homemade air bag containing 600–800 ml gas into the sternal gap, removing it 5 min later, blowing CO_2_ into the abdominal cavity, and maintaining it at 8mmHg. This method creates good surgical space, produces less chest infections, and helps achieve more satisfactory surgical results. Park et al. (23) found that robot-assisted single-hole thymectomy minimized the device collision problem caused by the subxiphoid approach and overcame the limitations of operation.

Jiang et al. and Liu et al. (24, 25) attempted a nonintubated subxiphoid uniportal video-assisted thoracoscopic thymectomy, which revealed lighter throat and tracheal injuries, manifested by reduced complications of postoperative dysphagia, sore throat, irritating cough, and so on. Nonintubated subxiphoid uniportal video-assisted thoracoscopic thymectomy was more consistent with the therapy of enhanced recovery after surgery (ERAS), aimed to reduce pain and hospital cost. A retrospective study was conducted by Mao et al. (26), in which a total of 40 patients underwent VATS thymectomy, with 21 patients undergoing nontracheal intubation (NI-VATS) and 19 tracheal intubation (I-VATS). Intraoperative and postoperative complications were similar in both groups, while the NI-VATS group had significant advantages in terms of anesthesia time, operative time, extubation time, total drainage volume, and postoperative pain scores.

Despite its many advantages, some drawbacks can still be listed for the subxiphoid approach. Lu et al. (27) noted some disadvantages of the subxiphoid approach: First, it's hard to separation tissue when severe adhesions exist. Second, the tumor size also needs to be limited because of the narrow space. Therefore, the application of the subxiphoid approach may be restricted in some operational conditions.

## The shortcomings of this study

There are some limitations and deficiencies in this study. First, this is a single-center retrospective study. Therefore, deviations are an inevitable fallout. A prospective study will be conducted to verify the conclusions drawn from this study. Second, the sample capacity of this study is relatively small, the time of the observation indicators is relatively short, and the relevant indicators are subjective, all of which will affect the statistical results. Finally, this study did not conduct propensity score matching analysis, which means it is prone to deviations.
