# Supplementary material for: Clinical analysis of subxiphoid vs. lateral approaches for treating early anterior mediastinal thymoma
Source: Front Surg. 2022 Sep 9;9:984043. doi: 10.3389/fsurg.2022.984043 (PMC9632990; doi:10.3389/fsurg.2022.984043)
Supplement: Supplementary file 1 [file Table1.docx]

|  | B | C | T/卡方 | P |
| --- | --- | --- | --- | --- |
| 性别 | 246 | 67 | .197 | .657 |
| 男 | 110 | 32 |  |  |
| 女 | 136 | 35 |  |  |
| 年龄 | 50.69±13.89 | 49.72±12.22 | .520 | .604 |
| BMI | 24.26±3.90 | 23.89±2.97 | .718 | .473 |
| 病灶直径 | 5.09±2.54 | 4.57±2.06 | 1.543 | .124 |
| 病理类型 |  |  | 11.384 | .044 |
| 胸腺瘤 | 51 | 17 |  |  |
| 胸腺囊肿 | 129 | 45 |  |  |
| 胸腺增生 | 33 | 2 |  |  |
| 胸腺癌 | 9 | 1 |  |  |
| 畸胎瘤 | 11 | 1 |  |  |
| 其他 | 13 | 1 |  |  |

一般情况比较

|  | B | C | T/卡方 | P |
| --- | --- | --- | --- | --- |
| 手术时间 | 98.71±42.47 | 77.81±29.93 | 4.595 | ＜0.01 |
| 术中出血量 | 56.87±82.01 | 34.70±34.38 | 3.305 | .001 |
| 中转开胸 | 2 | 0 |  |  |

术中情况比较

|  | B | C |  |  |
| --- | --- | --- | --- | --- |
| 术后引流量 | 359.01±461.74 | 219.40±270.90 | 3.152 | .002 |
| 置管时间 | 3.53±1.94 | 3.21±1.27 | 1.606 | .110 |
| 术后住院天数 | 4.40±2.09 | 3.91±1.42 | 2.247 | .026 |
| VAS | 4.59±0.79 | 4.42±0.78 | 1.577 | .116 |
| 非甾体消炎药 | 269.11±154.20 | 238.81±129.47 | 1.474 | .142 |
| 阿片类药物 |  |  | .040 | .842 |
| 是 | 172 | 46 |  |  |
| 否 | 74 | 21 |  |  |

术后情况比较
