# Supplementary material for: Clinical analysis of subxiphoid vs. lateral approaches for treating early anterior mediastinal thymoma
Source: Front Surg. 2022 Sep 9;9:984043. doi: 10.3389/fsurg.2022.984043 (PMC9632990; doi:10.3389/fsurg.2022.984043)
Supplement: Supplementary file 2 [file Table2.docx]

|  | A | C | T/卡方 | P |
| --- | --- | --- | --- | --- |
| 性别 | 99 | 67 | .225 | .635 |
| 男 | 51 | 32 |  |  |
| 女 | 48 | 35 |  |  |
| 年龄 | 49.05±12.56 | 49.72±12.22 | -.339 | .735 |
| BMI | 24.35±3.48 | 23.89±2.97 | .891 | .374 |
| 病灶直径 | 4.33±1.30 | 4.57±2.06 | -.840 | .403 |
| 病理类型 |  |  | 11.745 | .038 |
| 胸腺瘤 | 35 | 17 |  |  |
| 胸腺囊肿 | 42 | 45 |  |  |
| 胸腺增生 | 12 | 2 |  |  |
| 胸腺癌 | 3 | 1 |  |  |
| 畸胎瘤 | 5 | 1 |  |  |
| 其他 | 2 | 1 |  |  |

一般情况比较

|  | A | C | T/卡方 | P |
| --- | --- | --- | --- | --- |
| 手术时间 | 97.83±29.05 | 77.81±29.93 | 4.279 | ＜0.01 |
| 术中出血量 | 32.02±41.48 | 34.70±34.38 | -.554 | .663 |
| 中转开胸 | 0 | 0 |  |  |

术中情况比较

|  | A | C |  |  |
| --- | --- | --- | --- | --- |
| 术后引流量 | 240.03±169.54 | 219.40±270.90 | .554 | .581 |
| 置管时间 | 2.77±0.82 | 3.21±1.27 | -2.507 | .014 |
| 术后住院天数 | 3.75±1.02 | 3.91±1.42 | -.807 | .421 |
| VAS | 3.12±0.70 | 4.42±0.78 | -10.914 | ＜0.01 |
| 非甾体消炎药 | 228.28±148.49 | 238.81±129.47 | -.472 | .638 |
| 阿片类药物 |  |  | .018 | .894 |
| 是 | 67 | 46 |  |  |
| 否 | 32 | 21 |  |  |

术后情况比较
